# Editorial: Evolving Picture of Calcium Handling in Cardiac Disease

**DOI:** 10.3389/fphys.2020.01013

**Published:** 2020-09-04

**Authors:** Gema Ruiz-Hurtado, Angélica Rueda, Laetitia Pereira, María Fernández-Velasco

**Affiliations:** ^1^Cardiorenal Translational Laboratory, Institute of Research i+12, University Hospital 12 de Octubre, Madrid, Spain; ^2^CIBER-CV, University Hospital 12 de Octubre, Madrid, Spain; ^3^Department of Biochemistry, Center for Research and Advanced Studies of the National Polytechnic Institute (CINVESTAV-IPN), Mexico City, Mexico; ^4^UMR-S1180, Université Paris-Saclay, Châtenay-Malabry, France; ^5^La Paz University Hospital Health Research Institute, IdiPAZ, Madrid, Spain; ^6^CIBER-CV, Alberto Sols Biomedical Research Institute, Madrid, Spain

**Keywords:** calcium handling, ryanodine, heart disease, ion channels, EC coupling

Cardiovascular diseases (CVDs) underlie a high rate of mortality worldwide. Both acquired and inherited CVDs are closely related to ionic channelopathies, compromising the expression and function of a wide number of ion channels. Ca^2+^ mishandling has been associated with several CVDs, such as those involved in metabolic disorders, hypertrophy, heart failure, and several inherited heart diseases that implicate cardiac dysfunction and arrhythmias (Kushnir et al., 2018). Moreover, the archives of biomedical and life sciences are showing a multitude of contributions related to this topic, demonstrating the high interest of the scientific community in this specific research field. Indeed, Ca^2+^ is the key regulator of the Ca^2+^ induced Ca^2+^ release (CICR) process, the main actor in cardiac excitation-contraction coupling (ECC) (Fabiato, 1983). Briefly, ECC begins with membrane depolarization of cardiomyocytes, activating sarcolemmal L-type voltage-dependent Ca^2+^ channels, thus promoting an inward Ca^2+^ current (I_CaL_) that activates the intracellular Ca^2+^ channel/ryanodine receptor type 2 (RyR2), located at the sarcoplasmic reticulum (SR), triggering a transient increase of cytosolic Ca^2+^ levels, and eliciting cell contraction. To get new insights into these processes, Aguilar-Sanchez et al. evaluated in an elegant study whether the autonomous system regulates ECC in endocardial and epicardial layers from intact beating mouse hearts. The authors recorded action potentials (APs), Ca^2+^ transients, and Ca^2+^ currents in whole hearts, demonstrating that the Ca^2+^ influx occurring in AP phase 1, and not in phase 2, triggers ECC (Aguilar-Sanchez et al.).

After contraction, relaxation takes place when cytosolic Ca^2+^ levels return to physiological resting levels by two main mechanisms, the Sarco/Endoplasmic Reticulum Ca^2+^ ATPase (SERCA2a pump) and the sarcolemma Na^+^- Ca^2+^ exchanger (NCX) (Bers, 2002). More recently, other elements have been described as involved in the control of cardiac intracellular Ca^2+^ handling, such as transient receptor potential (TRP) channels or organelles such as the nucleus or mitochondria that also rely on transporters or channels for Ca^2+^ dynamics. The fine orchestration of all these elements results in adequate ECC and cell contraction.

Since its discovery (Inui et al., 1987), RyR2 has acquired special interest, and a large number of studies have analyzed whether changes in the structure, genetics, and function of this macro channel are involved in the pathogenesis of several CVDs. Post-translational modifications of RyR2, such as phosphorylation and oxidation, have gained major attention and have been related to channel dysfunction in some CVDs such as heart failure. Leading groups in the field have focused their interest in analyzing the physio-pathological regulation of RyR2 function by protein kinase A or calmodulin kinase type II-dependent phosphorylation. In this line, Federico et al. have performed an interesting review article that addresses the most relevant findings related to changes in SR-Ca^2+^ uptake and post-translational modifications in RyR2, such as the phosphorylation and oxidation both linked to arrhythmias and other cardiac disturbances. In the same line, Fernández-Miranda et al. demonstrated in a prediabetic rat model of metabolic syndrome an “*in situ”* impairment of RyR2 function elicited by reduced levels of RyR2 phosphorylation, together with depressed SR- Ca^2+^ uptake by SERCA2a, which are responsible for the poor cardiac outcome linked to this metabolic disorder.

In addition to phosphorylation, redox modifications of RyR2 such as S-nitrosylation and S-glutathionylation have emerged as new and potent modulators of channel function, demonstrating a crosslink between oxidation and phosphorylation changes. In many cases, changes in the redox state of RyR2 participate in the generation of abnormal Ca^2+^ diastolic release, supporting an additional pro-arrhythmogenic mechanism in several CVDs. Nikolaienko et al. recapitulate in a remarkable review article the most significant findings related to whether redox modifications of RyR2 can induce channel dysfunction. In this line, Hamilton et al. tested the efficacy of mitochondria-targeted pharmacological interventions to attenuate cardiac arrhythmia at the cellular level and in “*ex-vivo”* hypertrophied thoracic aortic-banded mouse hearts. The authors demonstrated that the abnormal mitochondrial Ca^2+^ accumulation induced an excessive production of reactive oxygen species that promoted the oxidation of RyR2 and enhanced its activity, inducing a pro-arrhythmic spontaneous Ca^2+^ release (Hamilton et al.).

Besides this, increased reactive oxygen species can also activate the inflammatory response. In many cases, pro-inflammatory mediators result in intracellular Ca^2+^ mishandling. In this regard, Val-Blasco et al. have determined whether the lack of the pro-inflammatory mediator NOD1 (a specific innate immune receptor) improves the β-adrenergic regulation of intracellular Ca^2+^ dynamics in failing hearts. NOD1 is a well-known activator of the inflammatory response. An increasing amount of evidences suggest that inflammatory mediators participate in the progression of CVDs (Adamo et al., 2020). Inflammation and metabolic disorders are closely related, and in many cases involve changes in cardiac Ca^2+^ homeostasis. A well-designed report, published by Delgado et al. has addressed the role of the inflammatory mediator tumor necrosis factor (TNF)-α in the modulation of intracellular Ca^2+^ handling in a mouse model of diabetes type II (*db/db*). The authors demonstrated a different regulation of Ca^2+^ handling mediated by TNF-α in male compared to female *db/db* mice, supporting the idea of a cardioprotective role of gender in the Ca^2+^ mishandling linked to a pro-inflammatory environment (Delgado et al.).

Another interesting contribution has been supported by Cagalinec et al. using a transgenic rat model with a mutation in the gene encoding the wolframin protein (*Wfs1*^−e5/−e5^), linked to diabetic and neurological complications. These authors demonstrated that *Wfs1*^−e5/−e5^ rats are euglycemic at 4 months old and ventricular cardiomyocytes isolated from *Wfs1*^−e5/−e5^ animals showed a higher duration of Ca^2+^ release; conjecturing a prolongation of RyR2 channel openings and modification in the RyR2 gating mediated by *Wfs1* in cardiac cells (Cagalinec et al.).

All these functional studies and reviews have contributed to a better understanding of the basis of CVDs arising from ion channel dysfunction and intracellular Ca^2+^ mishandling. These advances have also helped in the design of new drugs targeting ion channels, looking for more effective CVD treatments. Monsalvo-Villegas et al. performed an interesting pharmacological study describing a protective role of pirfenidone administration in isolated cardiomyocytes through modulation of CICR, ECC, and cell contractility. This study provides new explanations regarding the cardioprotective actions of pirfenidone (Monsalvo-Villegas et al.).

As previously mentioned, TRP channels have emerged as key partners in the pathogenesis of several CVDs, such as hypertrophy. Falcón et al. compiled in a commendable and well-organized review article the involvement of various subtypes of TRPs in cardiac physiology and CVDs. The authors particularly described the involvement of these receptors in heart remodeling linked to cardiac hypertrophy, uncovering a new and interesting field of research (Falcón et al.).

In addition to cardiac function, Ca^2+^ regulates vascular function. In this setting, Gutiérrez et al. have demonstrated in a well-conducted study whether different signaling pathways (PI3K, MAPK, and PKC) modulate Ca^2+^ entry and intracellular Ca^2+^ mobilization coupled to α_1_-adrenoceptor activation in the vascular smooth muscle of rat resistance arteries.

To conclude, the compendium of articles included in this Research Topic presents a new and interesting scenario for near future research related to novel actors in the picture of intracellular Ca^2+^ mishandling associated with CVDs.

## Author Contributions

All authors listed have made a substantial, direct and intellectual contribution to the work, and approved it for publication.

## Conflict of Interest

The authors declare that the research was conducted in the absence of any commercial or financial relationships that could be construed as a potential conflict of interest.
